# Machine Learning Model for Predicting Postoperative Complications in Pediatric Simple Congenital Heart Disease with Right Vertical Infra-Axillary Incision

**DOI:** 10.3390/jcdd13050208

**Published:** 2026-05-13

**Authors:** Chuli Shi, Yuehang Yang, Xinyi Liu, Hanshen Luo, Yongfeng Sun, Zhiwen Wang, Jiawei Shi

**Affiliations:** 1 Department of Cardiovascular Surgery, Union Hospital, Tongji Medical College, Huazhong University of Science and Technology, Wuhan 430022, China; 2 Department of Cardiovascular Surgery, Beijing Anzhen Hospital, Capital Medical University, Beijing 100029, China

**Keywords:** pediatric cardiology, machine learning, right vertical infra-axillary incision, postoperative complications, outcome

## Abstract

Background: This study aimed to develop and validate a machine learning model to predict postoperative complications in pediatric simple congenital heart disease (CHD) patients undergoing right vertical infra-axillary incision (RVIAI). Methods: A retrospective dataset of 638 patients who underwent treatment for ventricular septal defect and/or atrial septal defect via RVIAI at our hospital between August 2020 and August 2023 was collected. A total of 35 preoperative and intraoperative variables were used to construct 190 machine learning models. The optimal model was selected based on the highest mean C-index. Independent risk factors identified by the optimal model were ranked according to their importance. Kaplan–Meier analysis was used to compare the incidence of postoperative complications between different risk groups. Model performance was evaluated using the area under the receiver operating characteristic curve (ROC). Results: The optimal model, which combined Elastic Net (alpha = 0) and Gradient Boosting Machine, identified 18 baseline variables associated with postoperative complications. The top five predictors were defect size, globulin, activated partial thromboplastin time, red blood cell count, and blood urea nitrogen. Kaplan–Meier curves showed that postoperative complication rates were significantly higher in the high-risk group than in the low-risk group (*p* < 0.0001). The model demonstrated good discrimination, with area under the curve (AUC) values on postoperative days 5, 10, 15, and 20 remaining above 0.78 in both the training and test sets. Conclusions: This machine learning model provides a potential predictive tool for assessing postoperative risk in simple CHD patients undergoing RVIAI and may support more targeted perioperative management.

## 1. Introduction

Congenital heart diseases (CHDs) remain the most prevalent congenital anomalies in neonates, with reported incidence rates ranging from 0.4% to 5% among live births globally [1]. Despite marked reductions in postoperative mortality and reoperation rates achieved through advancements in surgical techniques, substantial challenges persist as postoperative complications continue to exert substantial impacts on survival outcomes within this vulnerable population [2,3]. Population-based analyses of multicenter data from U.S. healthcare institutions reveal that postoperative complication incidence rates in CHD patients range from 8.1% to 24.9%, with concurrent surgical mortality rates documented between 1.2% and 7.7% [4]. Additionally, long-term complication occurrence reduces survival rates over time as CHD patients age [5]. Therefore, in-hospital complication identification and targeted strategy development remain crucial for adverse event reduction in this population.

The Aristotle Basic Complexity (ABC) score was developed through multinational collaboration analyzing 18,024 surgeries across 32 centers to predict mortality/morbidity in 131 CHD subtypes [6]. While the Risk Adjustment for Congenital Heart Surgery-1 (RACHS-1) system demonstrates superior predictive validity for in-hospital mortality and length of stay compared to ABC [7], both systems exhibit two critical limitations: (1) excessive reliance on surgical parameters without incorporating preoperative patient factors, and (2) restriction to mortality prediction while omitting specific postoperative complications. Similarly, the Society of Thoracic Surgeons–European Association of Cardio-Thoracic Surgery Congenital Heart Surgery Mortality (STAT) score, a widely used risk stratification system that classifies operative risk based on procedural complexity, also centers primarily on mortality prediction and fails to address patient-specific postoperative complications [8]. These constraints reduce their clinical utility in preventing adverse postoperative outcomes.

The Cardiac Children’s Hospital Early Warning Score (C-CHEWS) has been developed to specifically predict postoperative conditions in pediatric cardiac patients, enabling prompt identification of cardiac arrest risks and unplanned ICU transfers to improve clinical outcomes [9]. However, this system’s partial dependence on subjective clinical assessments introduces potential variability in risk stratification accuracy. In contrast, machine learning-based predictive models utilize comprehensive clinical datasets—including demographic profiles, preoperative laboratory/imaging data, and intraoperative parameters—to objectively estimate individualized complication probabilities [10]. In this study, we employed over a hundred machine learning algorithms, leveraging patient demographic data, preoperative laboratory tests, preoperative imaging results, and intraoperative data for model development. The optimal predictive model was identified during the model development phase, with its reliability and clinical relevance subsequently confirmed via internal validation. This model aids in identifying postoperative complications in simple CHD patients and provides valuable guidance for early intervention and personalized treatment.

## 2. Materials and Methods

### 2.1. Study Design and Patients

**Figure 1:**
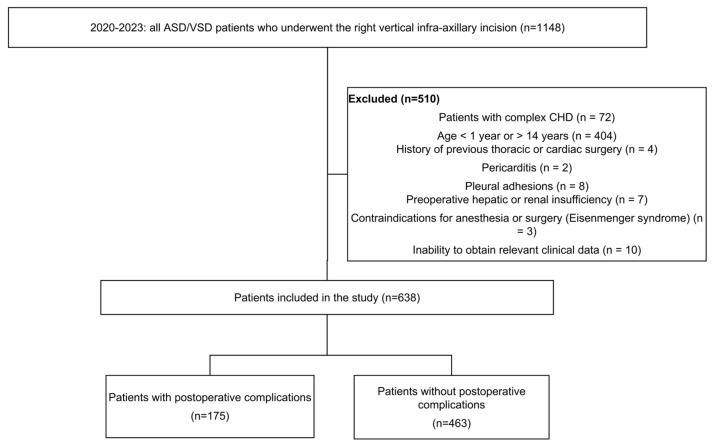
Flow chart for inclusion and exclusion of study participants.

We retrospectively reviewed 638 patients aged 1–14 years who underwent right vertical infra-axillary incision (RVIAI) for the treatment of atrial septal defect and/or ventricular septal defect (VSD) at our hospital between August 2020 and August 2023. All patients underwent color Doppler echocardiography to confirm the diagnosis. Inclusion criteria were as follows: (1) age between 1 and 14 years (this age range was selected to define a relatively homogeneous pediatric cohort for model development and to reduce age-related heterogeneity in anatomy, perioperative management, and postoperative risk); (2) definitive diagnosis of simple CHD (atrial septal defect and/or VSD) confirmed by color Doppler echocardiography. Exclusion criteria were as follows: (1) patients with complex CHD, (2) history of thoracic or cardiac surgery, (3) pericarditis, (4) pleural adhesions, (5) preoperative hepatic or renal insufficiency, (6) contraindications for anesthesia or surgery (Eisenmenger syndrome), (7) inability to obtain relevant clinical data (Figure 1).

This study was approved by the Ethics Committee of a large tertiary hospital in China (Approval No. 0035, 28 January 2024). The privacy of all subjects was reasonably protected, and all data were sourced from the inpatient medical records system. The study was conducted in accordance with the Declaration of Helsinki, and the need for informed consent was waived by the Ethics Committee due to the retrospective nature of the study.

### 2.2. Data Collection

Through electronic medical record review, including demographic variables (age, sex, height, and weight); laboratory measurements (red blood cell count [RBC], white blood cell count [WBC], hemoglobin [Hb], neutrophils, lymphocytes, total bilirubin, direct bilirubin, alanine aminotransferase [ALT], aspartate aminotransferase [AST], albumin, globulin, serum creatinine, blood urea nitrogen (BUN), uric acid, potassium, calcium, glomerular filtration rate, activated partial thromboplastin time [APTT], and international normalized ratio [INR]); and comorbidities (right ventricular outflow tract stenosis, mitral regurgitation, tricuspid regurgitation, pulmonary artery stenosis, left ventricular outflow tract stenosis, aortic regurgitation, pulmonary valve stenosis, and coronary artery anomalies). Intraoperative variables included operative time, cardiopulmonary bypass time, and aortic cross-clamp time. Postoperative evaluations included transthoracic echocardiography, chest radiography, and electrocardiography. Variables with more than 20% missing data were excluded, and those with less than 20% missing data were imputed using multiple imputations.

### 2.3. Study Composite Endpoint

The composite endpoint of this study was the occurrence of postoperative complications. All adverse events that occurred during the postoperative hospital stay were defined as postoperative complications: (1) Reoperation, (2) Subcutaneous emphysema, (3) Pneumothorax, (4) Pleural effusion, (5) Atrioventricular block, (6) Pericardial effusion, (7) Incision infection, (8) Atelectasis, (9) Pulmonary infection, and (10) Residual shunt. For the primary endpoint analysis, the event of interest was defined as the first occurrence of any prespecified postoperative complication (major or minor) during the index postoperative hospitalization.

### 2.4. Surgical Techniques

Patients underwent general anesthesia with endotracheal intubation and were placed in the left lateral decubitus position with the right arm elevated and supported to expose the axillary region. A 4–5 cm vertical main incision was made along the right midaxillary line from the 2nd to 4th intercostal space, designed to follow the natural muscle planes of the serratus anterior and intercostal muscles without transecting major muscle structures. An additional 0.5 cm vertical auxiliary incision was created in the 7th intercostal space along the midaxillary line for intraoperative cannulation assistance and postoperative chest tube placement. Thoracic access was gained through the 4th intercostal space. Single-lung ventilation of the left lung was performed to collapse the right lung, which was retracted posteriorly for full cardiac exposure. The thymus was dissected, and the pericardium was incised 2 cm anterior to the phrenic nerve, extending from the aorta to the inferior vena cava pericardial reflection. Standard ascending aortic and bicaval cannulation was performed, and cardiopulmonary bypass was initiated under mild hypothermia. After aortic cross-clamping, cardioplegic solution was administered via the aortic root to induce myocardial arrest. The right atrium was incised to expose the defect, which was repaired according to its anatomical type. A saline leak test was performed after repair, with any identified leaks sutured until complete sealing was confirmed. After rewarming and aortic cross-clamp release, the patient was weaned from cardiopulmonary bypass once stable cardiac function and circulation were restored. The right atriotomy was closed, and pericardial mediastinal drainage tubes were placed. Hemostasis was confirmed, and the chest was closed in layers with absorbable suture materials. All RVIAI procedures were performed by senior attending surgeons with ≥5 years of independent pediatric congenital heart surgery experience, who all followed a strictly standardized surgical protocol to minimize inter-surgeon procedural heterogeneity.

### 2.5. Development and Validation of Predictive Models

All patients were divided into a training set and a test set in a 7:3 ratio. Given the imbalance in outcome distribution, class-weighted learning was applied during model training, assigning higher misclassification costs to patients who developed postoperative complications. The training set was used for model construction, while the test set was used to assess model stability. This study was conducted under a time-to-event (survival analysis) framework, in which the outcome was defined as the time from surgery to the first postoperative complication. The time origin was defined as the date of surgery. Patients who did not develop postoperative complications during hospitalization were followed until hospital discharge and treated as censored at that time. Eight different models were used in this study, covering a variety of architectures and classification methods: Stepwise Cox regression (Step COX), Elastic Net (Enet; the optimal setting was alpha = 0, corresponding to ridge regression), Boost algorithm Cox regression (COXBoost), Random Survival Forest (RSF), Partial Least Square Regression (PLSR), Support Vector Machine (SVM), Gradient Boosting Machine (GBM), and Supervised Principal Component Analysis (Super PCA). We evaluated a total of 190 prespecified model combinations. These model combinations included both single-model approaches and sequential two-step combinations. In the two-step combinations, the first algorithm was used for feature selection or dimension reduction, and the second algorithm was used for final model fitting. In the optimal model combination, Elastic Net was applied in the first step for feature selection, and the selected variables were subsequently entered into the Gradient Boosting Machine for final model fitting. Feature importance was derived from the relative influence values of the final Gradient Boosting Machine model. To optimize model performance and reduce overfitting, five-fold cross-validation was conducted within the training set, with four folds used for model training and hyperparameter tuning and the remaining fold for internal validation. Hyperparameter tuning was performed using a grid search over predefined parameter ranges for each model combination. For each model combination, the optimal hyperparameter setting was selected based on the highest mean Harrell’s concordance index (C-index) across the validation folds. The final model combinations were then compared using the same cross-validation framework, and the combination with the highest mean C-index was selected as the optimal model. Kaplan–Meier survival curves were used to compare the incidence of postoperative complications between the high- and low-risk groups in both the training and test sets. Finally, time-dependent receiver operating characteristic (ROC) curves were constructed, and time-dependent area under the curve (AUC) values at postoperative days 5, 10, 15, and 20 were calculated to evaluate the discriminative performance of the model. The calibration ability of the model was evaluated using calibration plots and the Brier score in both the training and test sets.

### 2.6. Feature Scaling and Normalization

To ensure comparability across predictors and to meet the assumptions of scale-sensitive algorithms, all continuous variables were standardized prior to model development. Feature scaling was performed using z-score normalization based on the training dataset. The scaling parameters derived from the training set were subsequently applied unchanged to the test dataset.

### 2.7. Statistical Analysis

Continuous variables were expressed as mean ± standard deviation (SD) or median (Q25, Q75) for normally distributed data and as median with interquartile range (IQR) for non-normally distributed data. Comparisons between groups were made using the independent samples *t*-test or the Mann–Whitney U test. Categorical variables were reported as counts and percentages and analyzed using the chi-squared test or Fisher’s exact test. Statistical analyses were performed with SPSS Statistics for Windows, v27.0 (SPSS, Inc., Chicago, IL, USA). All tests were two-tailed, and *p* < 0.05 was considered statistically significant.

## 3. Results

### 3.1. Baseline Characteristics

This study included 638 patients diagnosed with atrial septal defect or ventricular septal defect, all of whom underwent surgery through a right vertical infra-axillary incision approach. Postoperative complications were observed in 162 patients (25.4% of the cohort). Complications included atelectasis in 62 patients, pneumothorax in 3, subcutaneous emphysema in 8, incision infection in 2, pericardial effusion in 5, atrioventricular block in 14 (all transient cases that resolved with conservative medical management, with no permanent atrioventricular block or pacemaker implantation), pleural effusion in 20, and two or more complications in 48 patients. Detailed baseline characteristics of all patients are shown in Table 1. After randomization, the cohort was divided into a training group of 448 patients and a test group of 190 patients (Supplementary Table S1). Significant differences between the two groups were observed in alanine aminotransferase and bilirubin levels (*p* < 0.05), while no significant differences were found in other baseline characteristics.

### 3.2. Model Construction

**Figure 2:**
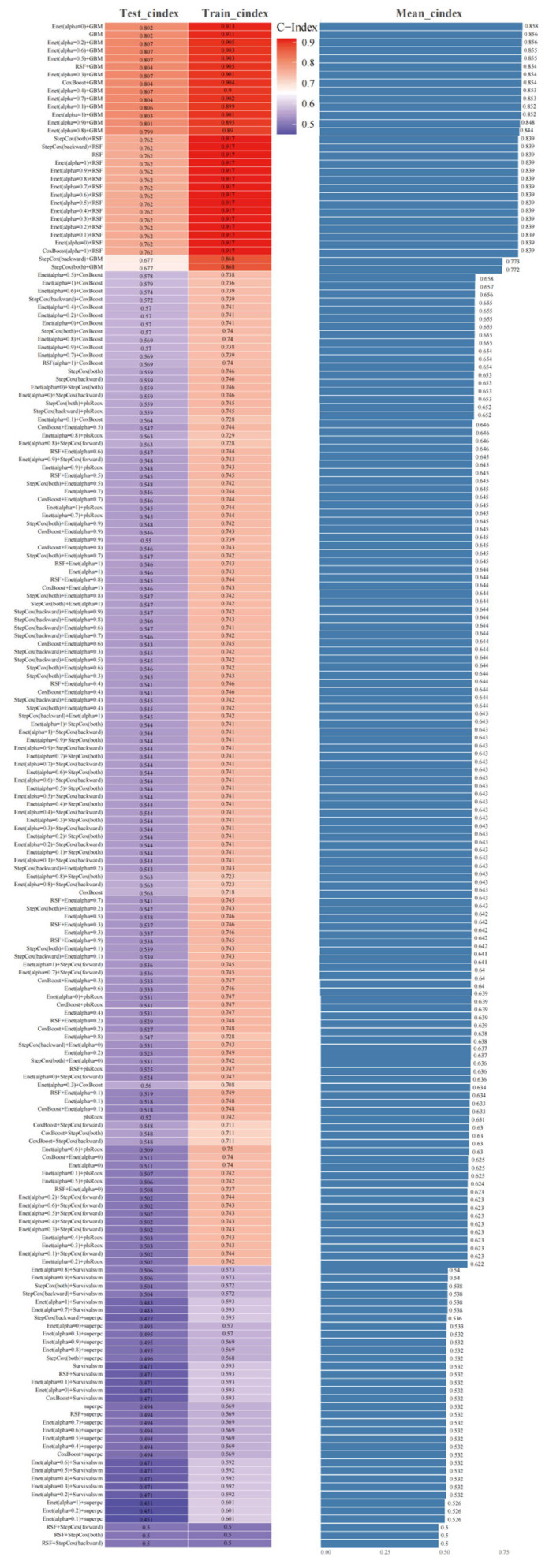
A total of 190 combinations of machine learning algorithms were developed, the C-index for each model was calculated, and the models were ranked by average C-index to determine the best predictive model.

A total of 190 machine learning model combinations were developed using the training set. Model training and hyperparameter optimization were performed within the training set using five-fold cross-validation, and the C-index across the five folds was used to compare model performance. Based on cross-validated performance, the model combining Elastic Net (Enet; alpha = 0) and GBM achieved the highest mean C-index (0.858) and was therefore selected as the optimal predictive model (Figure 2).

### 3.3. Feature Importance in the Model

**Figure 3:**
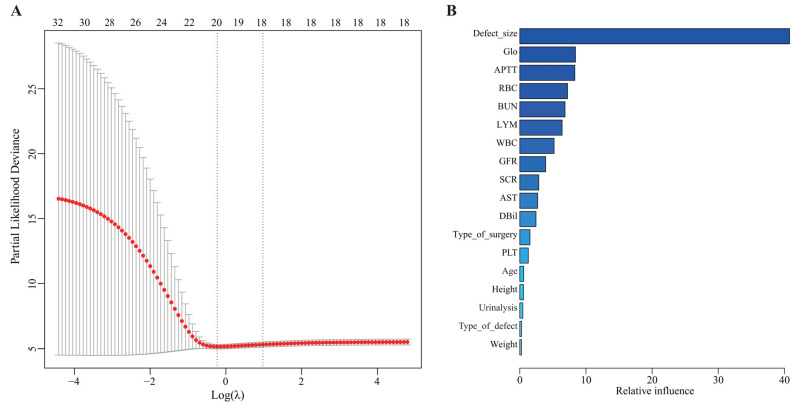
Important predictive features associated with postoperative complications in CHD patients. (**A**) Use Lasso regression to determine independent predictors of postoperative complications. (**B**) Rank the 18 independent predictors in descending order of relative influence.

The model combination of Enet (alpha = 0) and GBM identified 18 important predictive features for postoperative complications among 35 preoperative baseline characteristics in patients undergoing RVIAI (Figure 3A). These factors were then ranked in descending order of relative influence, including defect size, globulin, APTT, RBC, BUN, lymphocytes, white blood cells, glomerular filtration rate, serum creatinine, aspartate aminotransferase, direct bilirubin, type of surgery, platelets, age, height, urinalysis, defect type, and weight. Among these, defect size showed the highest relative importance in the model for predicting postoperative complications (Figure 3B).

### 3.4. Determining the Risk of Complications in Different Patient Groups

**Figure 4:**
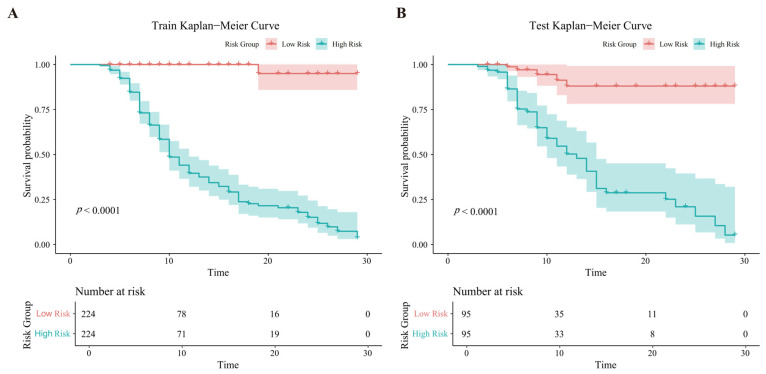
Kaplan–Meier curves for two groups with different risks of postoperative complications. (**A**) Kaplan–Meier curves for the training set, (**B**) Kaplan–Meier curves for the test set.

The patients were divided into high-risk and low-risk groups. In the training set, 448 patients were included, with 224 patients in each risk group. Kaplan–Meier analysis revealed that the incidence of postoperative complications was significantly lower in the low-risk group compared to the high-risk group (*p* < 0.0001) (Figure 4A). In the test set, 190 patients were included, with 95 patients in each risk group, and a similar statistically significant difference in complication incidence was observed between the two groups (*p* < 0.0001) (Figure 4B).

### 3.5. Model Performance

**Figure 5:**
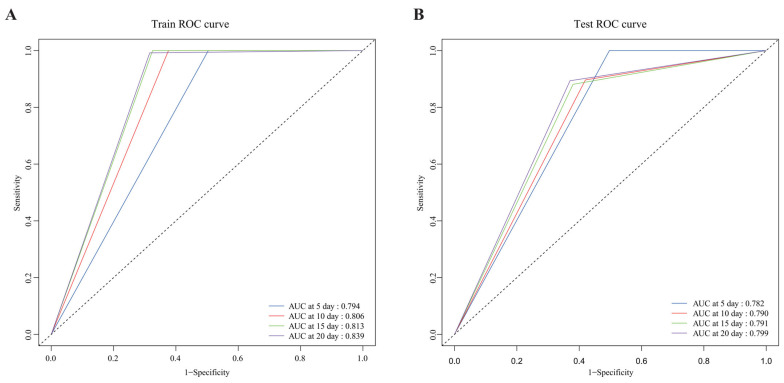
The predictive performance of the model was evaluated using the areas under the receiver operating characteristic (ROC) curve. The model demonstrated good performance in predicting postoperative complications on days 5, 10, 15, and 20 after surgery. (**A**) ROC curve for the training set, and (**B**) ROC curve for the test set.

**Figure 6:**
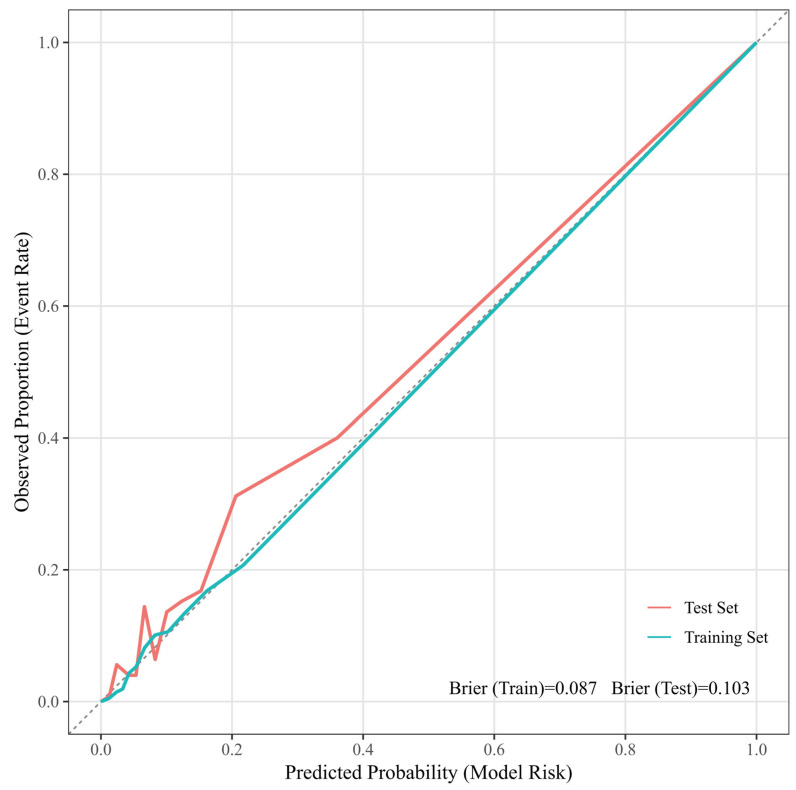
Calibration curves of the predictive model in the training and test sets.

To further evaluate the discriminative performance of the model, time-dependent ROC curves were generated using a set of 18 selected variables. In the training set, the time-dependent AUC values for postoperative days 5, 10, 15, and 20 were 0.794, 0.806, 0.813, and 0.839, respectively (Figure 5A). In the test set, the corresponding time-dependent AUC values for postoperative days 5, 10, 15, and 20 were 0.782, 0.790, 0.791, and 0.799, respectively (Figure 5B). In addition, the Brier scores were 0.087 in the training set and 0.103 in the test set (Figure 6).

## 4. Discussion

In this study, we developed 190 machine learning models to predict the risk of postoperative complications using demographic data, laboratory test results, and imaging features. The combination of Enet (alpha = 0) and GBM was identified as the optimal model, and was subsequently employed for validation.

Significant improvements in surgical techniques and perioperative management have resulted in extended survival duration among pediatric patients with CHD. However, mortality rates in this population remain elevated compared to age-matched general population cohorts [11]. Consequently, the preoperative identification of high-risk CHD patient cohorts is recognized as essential for reducing postoperative adverse outcomes. Compared with median sternotomy, RVIAI offers several notable advantages, such as reduced postoperative fluid output, shorter durations of mechanical ventilation, decreased hospital stay, and minimized injury to mammary and muscular tissues [12]. In our concurrent comparative cohort study at the same center, RVIAI showed postoperative outcomes comparable to those of median sternotomy and remained associated with fewer postoperative complications after multivariable adjustment [13]. Consistent with prior multicenter studies, these findings support that RVIAI is a safe and reliable surgical approach for the treatment of CHD, providing postoperative outcomes comparable to those of median sternotomy while offering superior cosmetic results. Consequently, RVIAI has gradually been recognized as a feasible alternative to traditional median sternotomy [14,15,16]. Nevertheless, postoperative complications remain difficult to avoid due to differences in patients’ preoperative and intraoperative conditions, which may still influence postoperative outcomes. To address this, we developed a machine learning prediction model to preoperatively identify high-risk CHD patients and prevent adverse postoperative outcomes. Multivariable analysis identified the following five predominant predictors, ranked by relative importance: (1) cardiac defect dimensions, (2) serum globulin concentrations, (3) APTT, (4) RBC, and (5) BUN.

Non-restrictive ventricular septal defects (VSDs) are pathologically characterized by extensive anatomical defects, frequently manifesting as substantial left-to-right shunting during early postnatal development. This hemodynamic abnormality predisposes patients to accelerated progression of pathophysiological complications. Concomitant nutritional deficiencies secondary to feeding impairment further exacerbate developmental delays, collectively contributing to elevated postoperative complication rates and protracted recovery trajectories [17]. Hemodynamic studies have demonstrated that larger VSD size is associated with significantly higher pulmonary arterial pressure and vascular resistance compared to restrictive defects [18]. Additionally, larger VSDs correlate with earlier onset of Eisenmenger syndrome, reflecting accelerated progression of right-to-left shunting [19]. These findings confirm the clinical significance of VSD size as an independent predictor of postoperative outcomes in congenital heart disease management.

Immunoglobulins exert anti-infective and anti-inflammatory effects by regulating inflammation and immune function within the body [20]. Following antigenic exposure, the production of immunoglobulins rapidly increases, initiating adaptive immunity and providing protective effects [21]. In pediatric CHD patients, chronic metabolic stress from elevated energy expenditure and recurrent pulmonary infections frequently induces immunoglobulin level elevation [22,23]. Patients experiencing postoperative complications typically show more severe inflammation compared to those without complications. This widespread inflammation can contribute to organ dysfunction, particularly affecting the kidneys through complex cellular processes [24]. Therefore, a potential synergistic relationship may exist between systemic inflammation, measured through immunoglobulin levels, and subclinical renal impairment, indicated by elevated BUN. This interaction could enable more precise risk stratification for postoperative complications.

Coagulation abnormalities, including deficiencies in specific clotting factors and von Willebrand factor, have been identified in CHD patients, potentially resulting from pathological shear stress patterns associated with uncorrected cardiac defects. This mechanistic hypothesis is supported by the normalization of coagulation parameters following surgical correction [25]. Furthermore, elevated von Willebrand factor expression in CHD patients with pulmonary hypertension has been linked to altered platelet function and adhesion dynamics [26]. The clinical significance of RBC counts demonstrates additional complexity: cyanotic CHD patients exhibit compensatory erythrocytosis, with mean RBC levels significantly exceeding reference ranges [27], while non-cyanotic CHD patients frequently demonstrate reduced RBC counts, potentially reflecting unique shunting-related mechanisms distinct from conventional anemia pathophysiology [28]. These findings collectively establish RBC count variability as a significant factor influencing postoperative complication risk in CHD patients. Preoperative elevations in RBC counts indicate heightened systemic inflammation, which may increase the susceptibility of cardiac surgical patients to postoperative infections and adverse cardiovascular events [29]. In addition, prior studies have reported that a higher preoperative neutrophil-to-lymphocyte ratio is associated with the development of low cardiac output syndrome in children undergoing surgery for congenital heart disease [30]. These findings suggest that heightened preoperative inflammatory activity may contribute to poorer postoperative outcomes in children with CHD. Platelet reserves in CHD patients may be depleted as a consequence of sustained inflammatory stimulation, which can increase the risk of postoperative bleeding [31]. Moreover, exposure of circulating blood to extensive artificial surfaces during cardiopulmonary bypass leads to pronounced activation of platelets and coagulation pathways, predisposing CHD patients to postoperative thrombotic events [32]. Moreover, intraoperative mechanical shear stress triggers the release of inflammatory mediators, leading to postoperative coagulopathy and systemic inflammatory response [33]. Therefore, preoperative assessment of these factors may help identify high-risk individuals and guide targeted perioperative management to improve postoperative outcomes in CHD patients.

Body weight is a critical determinant of perioperative technical complexity and complication risk in pediatric congenital heart surgery. In our institutional practice, RVIAI remains safe and effective for simple CHD across the full weight range of our 1–14 years cohort, with extreme body weight not considered an absolute contraindication. This practice is supported by robust clinical evidence: a 15-year study of 1126 patients confirmed the safety of RVIAI across a body weight range of 6 to 88 kg, and further demonstrated that a body mass index up to 47.83 kg/m^2^ was not a contraindication for this approach, with favorable short- and long-term survival outcomes [16]. For low-weight infants ≤5 kg, RVIAI achieved comparable perioperative safety to median sternotomy, with reduced surgical trauma and faster postoperative recovery [34]. While these cumulative data confirm the overall feasibility and safety of RVIAI across broad weight strata, extreme body weight increases the difficulty of surgical exposure and perioperative hemodynamic management, which may indirectly elevate the risk of postoperative complications. This clinical relevance supports the inclusion of weight and height as predictive features in our final model. From a clinical perspective, this model is intended to function as a preoperative risk stratification tool. Patients identified as high risk may benefit from intensified preoperative evaluation, targeted optimization of inflammatory and hematologic abnormalities, and perioperative surveillance. Importantly, the model is designed to support clinical decision-making, rather than to replace clinician judgment.

The temporal progression of AUC values (0.784–0.841 across postoperative days 5–20) implies increasing model discriminative capacity with longer follow-up, potentially reflecting delayed manifestations of subclinical complications. Clinical utility was further validated by the Kaplan–Meier analysis, which showed significantly higher rates of postoperative complications in the high-risk patient group compared with the low-risk patient group, supporting risk stratification for targeted perioperative surveillance. By incorporating common CHD-specific postoperative complications as primary endpoints, this model enables early identification of high-risk patients during clinical management, potentially mitigating progression to severe adverse outcomes. As RVIAI approaches gain increasing adoption for complex CHD repair, model validation across diverse anatomical variations will be essential to ensure broad clinical applicability [35]. Furthermore, the temporal variability of laboratory parameters (e.g., fluctuations in immunoglobulin levels following recent infections) presents inherent challenges to static predictive frameworks. Future efforts could integrate longitudinal data streams from wearable sensors monitoring inflammatory biomarkers such as interleukin-6 to enable dynamic risk stratification.

## 5. Limitations

This study has several limitations. First, the composite primary endpoint of this study includes postoperative adverse events with markedly heterogeneous clinical severity. Stratified risk prediction for complications of different severity will be explored in future studies with expanded sample sizes. Second, this is a single-center retrospective study with only internal validation, so the generalizability of the proposed model needs to be further confirmed by multicenter external validation. Third, surgeon-related variables were not included in this preoperative prediction model, though all procedures were performed by standardized senior attending surgeons. Finally, because the study was restricted to patients aged 1–14 years to improve cohort homogeneity, the generalizability of the model to infants younger than 1 year and to older adolescents should be interpreted with caution.

## 6. Conclusions

This study developed a new predictive model to assess the risk of postoperative complications in simple CHD patients undergoing RVIAI approach, identifying key predictive factors for postoperative complications, such as defect size, globulin, APTT, and BUN. The model may help stratify postoperative risk in this population; however, its potential clinical application should be interpreted with caution and requires further external and prospective validation.

## Figures and Tables

**Table 1:** Demographic characteristics of patients.

Characteristics	Complication (*n* = 162)	Non-Complication (*n* = 476)	*p* Value
Age, year	3 (1, 6)	2 (1, 4)	0.001
Males, gender	78 (48.1%)	221 (46.4%)	0.795
BMI, kg/m^2^	15 (14, 17)	15 (14, 17)	0.682
hospital stay, day	13.0 (10.5, 14.5)	13.0 (11.0, 15.0)	0.565
Classification of CHD			0.004
VSD	55 (34.0%)	204 (42.9%)	
ASD	76 (46.9%)	139 (29.2%)	
VSD + ASD	31 (19.1%)	133 (27.9%)	
Location of VSD			0.049
Inlet	4 (7.3%)	18 (8.8%)	
Outlet	6 (10.9%)	35 (17.2%)	
Double-committed	4 (7.3%)	6 (2.9%)	
Peri-membranous	41 (74.5%)	145 (71.1%)	
Preoperative comorbidities			0.107
Mitral regurgitation	4 (2.5%)	18 (3.8%)	
Tricuspid regurgitation	50 (30.9%)	91 (19.1%)	
Pulmonary valve stenosis	1 (0.6%)	11 (2.3%)	
Pulmonary artery stenosis	0 (0%)	2 (0.4%)	
RVOTS	3 (1.9%)	13 (2.7%)	
LVOTS	1 (0.6%)	2 (0.4%)	
Coronary sinus aneurysm	0 (0%)	3 (0.6%)	
Aortic valve insufficiency	1 (0.6%)	8 (1.7%)	
At least two complications	6 (3.7%)	31 (6.5%)	
Defect size, cm^2^	0.4 (0.3, 1.2)	0.8 (0.6, 1.5)	<0.001
Pulmonary hypertension	14 (8.0%)	40 (8.6%)	0.921
LVEF, %	65 (63, 66)	65 (64, 66)	0.676
Red blood cell	4.47 (4.25, 4.82)	4.53 (4.43, 4.77)	0.322
White blood cell	7.58 (6.23, 9.19)	7.79 (6.35, 9.24)	0.841
Hemoglobin	123.58 ± 10.16	124.62 ± 10.38	0.018
Blood platelet	291.0 (255.0, 324.5)	292.0 (261.0, 355.0)	<0.001
Neutrophils	30.7 (23.38, 42.23)	31.2 (25.32, 40.85)	0.294
Lymphocyte	58 (45.1, 65.5)	55.4 (48.6, 61.2)	0.408
Total bilirubin	7.5 (5.6, 9.8)	7.0 (5.5, 9.4)	0.089
Direct bilirubin	2.5 (1.8, 3.6)	2.2 (1.8, 3.4)	0.028
Alanine aminotransferase	16.0 (13.5, 22.0)	17.0 (13.5, 21.0)	0.952
Aspartate aminotransferase	36.0 (30.0, 43.0)	40.0 (32.5, 45.0)	0.231
Albumin	43.1 (41.3, 44.6)	43.2 (41.8, 44.8)	0.022
Globulin	20.9 ± 4.1	20.4 ± 3.9	<0.001
Serum creatinine	29.1 (24.6, 36.8)	27.7 (24.1, 32.2)	<0.001
Blood urea nitrogen	4.53 (3.90, 5.31)	4.43 (3.93, 4.89)	0.001
Uric acid	259.0 (222.2, 301.1)	245.8 (219.3, 284.2)	0.176
Kalium	4.55 (4.33, 4.92)	4.55 (4.35, 4.76)	0.427
Calcium	2.42 ± 0.13	2.44 ± 0.11	0.215
Glomerular filtration rate	189.88 ± 29.21	190.12 ± 29.98	<0.001
APTT	38.0 (35.62, 41.27)	40.3 (38.2, 42.4)	0.001
INR	1.05 (0.99, 1.11)	1.05 (0.98, 1.09)	0.939

Measures were expressed as mean ± SD and median (Q25, Q75), and counts are expressed as *n* (%); continuous variables were compared between groups using t-test or the Mann–Whitney U test, and categorical variables were analyzed using the chi-squared test or Fisher’s exact test. Abbreviations: BMI, body mass index; CHD, congenital heart disease; VSD, ventricular septal defect; ASD, atrial septal defect; RVOTS, right ventricular outflow tract stenosis; LVOTS, left ventricular outflow tract stenosis; LVEF, left ventricular ejection fraction; APTT, activated partial thromboplastin time; INR, international normalized ratio.

## Data Availability

The data supporting this study’s findings are available from the corresponding author upon reasonable request.

## Supplementary Materials

The following supporting information can be downloaded at: https://www.mdpi.com/article/10.3390/jcdd13050208/s1, Table S1: Clinical variables included in the machine learning model across training and test sets.
